# COVID-19 hospitalization rates rise exponentially with age, inversely proportional to thymic T-cell production

**DOI:** 10.1098/rsif.2020.0982

**Published:** 2021-03-17

**Authors:** Sam Palmer, Nik Cunniffe, Ruairí Donnelly

**Affiliations:** ^1^ Mathematical Institute, University of Oxford, Oxford, UK; ^2^ Department of Plant Sciences, University of Cambridge, Cambridge, UK

**Keywords:** thymus, T-cell, immunology

## Abstract

Here, we report that COVID-19 hospitalization rates follow an exponential relationship with age, doubling for every 16 years of age or equivalently increasing by 4.5% per year of life (*R*^2^ = 0.98). This mirrors the well-studied exponential decline of both thymus volume and T-cell production, which halve every 16 years. COVID-19 can therefore be added to the list of other diseases with this property, including those caused by methicillin-resistant *Staphylococcus aureus*, MERS-CoV, West Nile virus, *Streptococcus pneumoniae* and certain cancers, such as chronic myeloid leukaemia and brain cancers. In addition, the incidence of severe disease and mortality due to COVID-19 are both higher in men, consistent with the degree to which thymic involution (and the decrease in T-cell production with age) is more severe in men compared to women. Since these properties are shared with some non-contagious diseases, we hypothesized that the age dependence does not come from social-mixing patterns, i.e. that the probability of hospitalization *given infection* rises exponentially, doubling every 16 years. A Bayesian analysis of daily hospitalizations, incorporating contact matrices, found that this relationship holds for every age group except for the under 20s. While older adults have fewer contacts than young adults, our analysis suggests that there is an approximate cancellation between the effects of fewer contacts for the elderly and higher infectiousness due to a higher probability of developing severe disease. Our model fitting suggests under 20s have 49–75% additional immune protection beyond that predicted by strong thymus function alone, consistent with increased juvenile cross-immunity from other viruses. We found no evidence for differences between age groups in susceptibility to infection or infectiousness to others (given disease state), i.e. the only important factor in the age dependence of hospitalization rates is the probability of hospitalization given infection. These findings suggest the existence of a T-cell exhaustion threshold, proportional to thymic output and that clonal expansion of peripheral T-cells does not affect disease risk. The strikingly simple inverse relationship between risk and thymic T-cell output adds to the evidence that thymic involution is an important factor in the decline of the immune system with age and may also be an important clue in understanding disease progression, not just for COVID-19 but other diseases as well.

## 1. Introduction

Epidemiological patterns in the incidence of a disease can provide insight into the mechanisms of disease progression [1–4]. The degradation of the adaptive immune system with age is already acknowledged to be a major risk factor for both infectious and non-infectious diseases and may play a role in understanding the emerging COVID-19 epidemic. Thymus volume, and the concomitant production of T-cells, decrease exponentially with age with a half-life of 16 years, or equivalently by 4.5% per year [5,6] (electronic supplementary material, figure S3*a*). These changes in the adaptive immune system contribute to less robust immune responses in elderly individuals [7]. In this paper, we analyse age and sex trends in national COVID-19 hospitalization data, in order to investigate the role of immune function in the ongoing coronavirus pandemic.

COVID-19 disease progression can be characterized by three consecutive phases of increasing severity [8,9]. First, there are mild symptoms such as a dry cough, sore throat and fever. After this point, the majority of cases will undergo spontaneous regression [10]. Second, some patients can develop viral pneumonia, requiring hospitalization [8]. The third stage, typically occurring three weeks after the onset of symptoms, is characterized by fibrosis [8] and leads to life-threatening symptoms [10–12]. COVID-19 patients often exhibit lymphopenia, i.e. extremely low blood T-cell levels, even in the first few days after the onset of symptoms, which is a predictor of disease progression and mortality [13,14]. Clinical trials are currently underway to test T-cell-based immunotherapies [15,16] and vaccines that elicit T-cell, as well as antibody, responses [17]. There is evidence that T-cells may be more effective than antibodies as exposed, asymptomatic individuals develop a robust T-cell response without (or before) a measurable humoral response [18].

The relationship between COVID-19 risk and age has been extensively explored [19–22], and age-stratified, contact-based, transmission models have accurately explained various aspects of the pandemic [20,21,23,24]. In particular, these studies have found that the risk of severe disease rises with age and is especially low for those under 20. Some studies suggest that non-adults are as likely to be infected as adults, but then have lower risk of disease progression [24] while others find lower risk of both infection and disease progression in the under 20s [21,23]. While these studies have looked at COVID-19 risk and age, here we go further by relating these trends to thymic involution and T-cell production. This may lead to a mechanistic understanding of disease progression.

Several diseases have risk profiles that increase exponentially with age, doubling every 16 years, i.e. risk is proportional to e^0.044*t*^, where *t* is age, or equivalently increasing by about 4.5% per year [4]. These diseases are caused by a range of pathogens, from bacterial (methicillin-resistant *Staphylococcus aureus* (MRSA), *S. pneumoniae*) to viral (West Nile virus, MERS-CoV [25]) and even include some cancers (chronic myeloid leukaemia, heart and brain cancers). Since thymus volume and T-cell production both decrease with age exponentially, halving every 16 years [5], disease risk is therefore inversely proportional to T-cell production for these diseases. A mechanistic model has been proposed to explain this inverse relationship, incorporating an immune escape threshold and stochastic fluctuations in antigen levels [4]. Furthermore, the sex bias in thymic involution (and T-cell production) also roughly matches the sex bias in disease risk, with men having approximately 1.3–1.5 times higher overall cancer and infectious disease risk [26–28] and approximately 1.5 ± 0.3 times lower T-cell production, as measured by T-cell receptor excision circles (TRECs), a proxy for thymic output [4,6]. As such, fundamental patterns in disease incidence with respect to both age and sex can be directly linked to differences in the adaptive immune system. We therefore tested to see if COVID-19 follows the same trend.

## 2. Results

### 2.1. COVID-19 hospitalization rates

**Figure 1.**
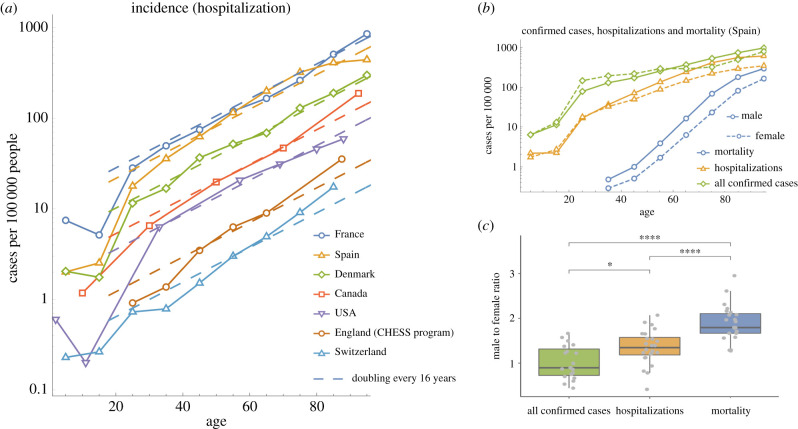
(*a*) For adults, incidence of COVID-19 hospitalizations rises exponentially with age, doubling with every 16 years of age. See electronic supplementary material, table S3, for a full list of data sources. (*b*) Data from Spain on all confirmed cases, hospitalizations and mortality, from a single study early in the epidemic, show a sex bias which increases with disease severity. (*c*) Boxplot showing male to female ratios for incidence, hospitalization rates and mortality, across all age groups with non-zero entries, from the following countries: France, England, Wales and Spain.

While data on confirmed cases can be highly variable and largely influenced by testing strategies, the data on hospitalizations, which is the focus of this paper, are relatively more reliable. The incidence of COVID-19 hospitalizations, in a number of countries, consistently doubles with every 16 years of age (*R*^2^ = 0.98 for top three countries; figure 1*a*). Meanwhile, the incidence of all confirmed cases (including mild or asymptomatic) appears roughly constant across adult ages (electronic supplementary material, figure S1). One explanation that is consistent with the data is that exposure is approximately uniform for adult age groups and that after exposure, the probability of becoming hospitalized is proportional to e^0.044*t*^, where *t* is age. We will address the age dependence of exposure in more detail by accounting for assortative social mixing as well as a range of additional age-dependent factors in our Bayesian model (see below).

There is a sex bias in COVID-19 risk, which increases with disease severity (figure 1*c*). This is similar to other diseases, including cancer, where men have 1.33 times the risk of hospitalization and 1.89 times the risk of death [26,29]. The sex bias in COVID-19 is remarkably similar to a factor of 1.35 ± 0.4 for hospitalization incidence and 1.9 ± 0.4 for mortality (mean ± s.d.; figure 1*c*). The slope of the logarithm of the COVID-19 mortality curve is over twice that of the hospitalization curve, corresponding to an exponential with rate 0.109 ± 0.005 yr^−1^ (figure 1*b*). Another way of thinking about the sex bias would be to say that for both hospital incidence and mortality, men are effectively approximately 6 years older than women in terms of risk. Other risk factors such as BMI can also be viewed similarly to give an individualized effective ‘Covid age' [30]. The increase in mortality with age may also be explained by comorbidities which increase with age, such as cardiovascular disease, which rises exponentially [31] with a rate of 0.071 ± 0.003 yr^−1^. Since 0.071 + 0.044 = 0.115 ≈ 0.109, a simple model where the risk of COVID-19 mortality is proportional to the risk of cardiovascular disease and inversely proportional to T-cell production would have the correct age dependence. This would suggest that cardiovascular disease is a risk factor just for the stages in between hospitalization and death.

### 2.2. Bayesian model

Similar to other diseases, COVID-19 hospitalization risk is relatively high for very young children (e.g. 0.6 cases per 100 000 for ages 0–4 versus 0.2 cases per 100 000 for ages 5–17 in USA; figure 1*a*). Additionally, older children have a risk lower than expected based on the exponential increase with age we have identified (figure 1*a*). This is similar to MRSA and *S. pneumoniae* infection, but not West Nile virus infection or cancers with similar exponential behaviour [4]. Potential factors underlying the apparent low risk in juveniles include age dependence in (i) exposure (e.g. due to heterogeneous social mixing among age groups), (ii) disease progression, (iii) infection given exposure and/or, (iv) infectiousness to others. Throughout this paper, we use the term ‘severe infection' synonymously with hospitalization and we categorize all infections as either mild or severe. In a preliminary analysis, we first incorporated contact matrices into a simple analytically tractable susceptible–infected–removed (SIR) model to predict the steady state of the age distribution of hospitalizations in France, with the assumption that the probability of severe disease given infection is proportional to e^0.044*t*^ (see electronic supplementary material, figure S3). This model suggested that age differences in social mixing could, in part, account for the relatively low hospitalization of non-adults (electronic supplementary material, figure S3). However, the other possible factors in low juvenile COVID-19 hospitalization were not considered in this preliminary analysis.

To incorporate all relevant factors, and to rigorously test the hypothesis that the probability of hospitalization given infection rises with age at the same rate as thymic involution, we conducted a more detailed analysis of age dependence based on daily hospitalization, recovery and death data. We focused on the single country France, for which an unusually comprehensive age distributed dataset is available [20]. All cases in the dataset are either biologically confirmed or present with a computed tomographic image highly suggestive of SARS-CoV-2 infection, and the dataset includes corrections for reporting delays [20]. We formulated an age-structured Bayesian SIR model of infection, partitioning the force of infection into that arising from contacts with mild and severely infected individuals, weighted by age-dependent contact matrices, as well as contact-independent (environmental) transmission. The model-fitting exercise focused on inferring a posterior parameter distribution for the probability of severe disease given infection for each age cohort. In addition, posterior distributions were inferred for a range of secondary parameters (electronic supplementary material, table S2, parameters of the Bayesian analysis), including age-dependent transmissibility and susceptibility.

**Figure 2.**
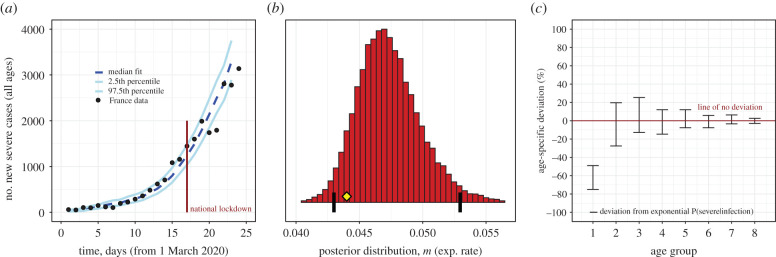
(*a*) Forward simulation of the French epidemic using the fitted parameters (*b*,*c*) produces a credible interval containing the French hospitalization data up to day 24. (*b*) The 95% credible interval for the rate of age-dependent exponential growth in hospitalization probability includes the rate of thymus degradation (0.044 years^−1^, yellow diamond). Black vertical lines show the 2.5th and 97.5th percentiles. (*c*) The juvenile cohort has additional significant protection beyond what is predicted by their stronger thymus function (red interval is separated from the zero deviation line for juveniles only). See electronic supplementary material, ‘Bayesian modelling' for full description of methods.

Our results reiterate that the probability of severe disease given infection increases exponentially with age, at a rate that is remarkably well matched by the rate of thymus decline for all age groups above 20 years (electronic supplementary material, figure S4; all adult age groups have 95% credible intervals including the rate of thymus decline). In order to investigate the nature of juvenile deviation from this exponential relationship, we reformulated the analysis to allow deviations from an exponential increase (for the probability of severe disease given infection) for each age cohort (figure 2). The posterior parameter distribution for the exponential rate was found to match the rate of thymic degradation (95% CI 0.043–0.053 yr^−1^; figure 2*b*). Only the juvenile age cohort was found to significantly deviate from the exponential response (figure 2*c*), showing a level of additional protection to severe COVID-19 of between 49 and 75% (electronic supplementary material, table S1). Our sensitivity analysis allowed—within each age cohort—for deviation from uniform the probability of infection given exposure, and, deviation from uniform infectiousness of infected individuals. For both of these, we found that none of the age cohorts deviated significantly (in all cases 95% credible intervals included zero deviation; electronic supplementary material, figure S5), allowing us to discount these potentially confounding factors.

The low susceptibility to severe disease given infection in non-adults may be due to cross-protection from other coronaviruses [8,32–34], or even non-specific protection from other respiratory viruses [35], which occur more frequently in non-adults compared to adults [36]. Our estimate of 49–75% protection ties in with a study which found SARS-CoV-2 reactive antibodies in approximately 60% of unexposed individuals aged 6–16 and only 6% in adults [37]. There is also evidence of unexposed individuals having SARS-CoV-2 reactive CD4+ T-cells [38]. Another possible explanation for the low risk in non-adults might come from some intrinsic feature of the immune system. For example, we speculate that the cause may be related to the high risk of T-lymphoblastic leukaemia for ages 5–20 (see [4]; electronic supplementary material, figure S5).

## 3. Discussion

Although we have demonstrated a clear relationship between the probability of severe disease and age, it is possible that the relationship is due, in part, to alternative physical processes other than T-cell production, such as age-related changes in the bone marrow, spleen or lymph nodes. Bone marrow also shrinks with age, but at a rate that is substantially slower than the thymus [39]. Further experiments are needed to determine the degree of causation between T-cell production and disease risk, for example, by measuring TRECs and performing a prospective study or quantifying the increase in risk in thymectomized individuals [40]. Furthermore, a mechanism for why the probability of hospitalization is inversely proportional to T-cell production is currently lacking. One possible model features stochastic fluctuations in the number of infected cells and an immune escape threshold which is proportional to T-cell production [4]. This model has the added benefit that it can also explain most of the other (non-exponential) relationships between risk and age seen in various cancer types [4].

Chronic myeloid leukaemia (CML) is a type of cancer with an age dependence remarkably similar to COVID-19. In both diseases, the risk of hospitalization rises exponentially, inversely proportional to T-cell production [4], with sex bias ratios of 1.35 ± 0.4 for COVID-19 and 1.35 ± 0.3 for CML. The mortality risk profiles are also similar (exponential rates: 0.109 ± 0.005 yr^−1^ for COVID-19 and 0.103 ± 0.007 yr^−1^ for CML; sex bias ratios: 1.9 ± 0.4 for COVID-19 and 1.8 ± 0.6 for CML; electronic supplementary material, figure S2). CML is characterized by a single genomic feature, a chromosomal translocation known as the Philadelphia chromosome. This suggests that the probabilities of Philadelphia chromosome formation and COVID-19 infection are approximately age independent, but that the probabilities of subsequent hospitalization are T-cell dependent. A good candidate for a potential mechanism involves the phenomenon that increased antigenic load can lead to T-cell exhaustion, characterized by low effector function and clone-specific depletion [41]. T-cell exhaustion is a factor in both cancer and infectious diseases, including COVID-19 [42,43], where it has even been shown to be a predictor of mortality [44]. As T-cell production decreases with age, this may lead to an increase in the probability of T-cell exhaustion. In support of this hypothesis, low precursor T-cell numbers have been shown to lead to T-cell exhaustion and disease progression in a mouse cancer model [45]. More specifically, we predict a step in disease progression with a probability exactly inversely proportional to the number of precursor T-cells.

When looking at sex biases for COVID-19 hospitalization and mortality (figure 1*c*), we found factors of 1.35 and 1.9, respectively. We can speculate that since 1.35^2^ ≈ 1.9, this might be an indication that among the steps of disease progression, there could be two T-cell-dependent steps, one pre-hospitalization and one post-hospitalization. This would imply that the risk of death would involve two factors of e^0.044*t*^ and therefore mortality would increase at least as fast as e^0.088*t*^. The log-slope of the mortality curve being 0.109 ± 0.005 yr^−1^ is consistent with this hypothesis. One feature of post-hospitalization disease progression is an IL-6 driven cytokine storm [46], which has been related to T-cell dysfunction in a mouse model [47]. These T-cell dysfunction-related cytokine storms were attenuated by nicotinamide adenine dinucleotide precursors and blocking of TNF*α* signalling.

## 4. Conclusion

Here, we have shown that the risk of COVID-19 hospitalization rises exponentially with age, inversely proportional to T-cell production, in a similar way to several other diseases. Consistently, the sex bias in disease risk also fits this trend. These features suggest that the risk of hospitalization is related to an immune deficiency, rather than an immunopathology. By contrast, long-COVID follows patterns similar to autoimmune diseases, with middle-aged women having the highest risk [48]. In addition, we found that the under-20 age group benefits from additional protection from severe disease by a factor similar to the prevalence of SARS-CoV-2 cross-reactive antibodies. Our mathematical model suggests that the age dependence of hospitalization rates does not arise from differences in social-mixing patterns, but rather from the probability of hospitalization given infection. The model could be easily extended to assess which age groups and socioeconomic groups would be most valuable to vaccinate and therefore to optimize vaccination strategies.

These findings add to the growing evidence that thymic involution is a major component of immunosenescence and that restoring thymus function may be an effective preventative measure for many common diseases. Additionally, our understanding of host–pathogen dynamics is not complete. There is currently no detailed mechanistic model to explain why the probability of hospitalization would be proportional to the reciprocal of thymic T-cell production, for COVID-19 or any other disease. We hope that these findings will be an important clue in understanding the precise mechanisms involved in disease progression.

## Data accessibility

All data and code are available in the main text or the electronic supplementary material, or at https://github.com/samIndeed/COVID.

## References

[1] Armitage P, Doll R. 2004 The age distribution of cancer and a multi-stage theory of carcinogenesis. Br. J. Cancer **91**, 1983-1989. (10.1038/sj.bjc.6602297) 15599380 PMC2410148

[2] Tomasetti C, Vogelstein B. 2015 Cancer etiology. Variation in cancer risk among tissues can be explained by the number of stem cell divisions. Science **347**, 78-81. (10.1126/science.1260825) 25554788 PMC4446723

[3] Tomasetti C, Marchionni L, Nowak MA, Parmigiani G, Vogelstein B. 2015 Only three driver gene mutations are required for the development of lung and colorectal cancers. Proc. Natl Acad. Sci. USA **112**, 118-123. (10.1073/pnas.1421839112) 25535351 PMC4291633

[4] Palmer S, Albergante L, Blackburn CC, Newman TJ. 2018 Thymic involution and rising disease incidence with age. Proc. Natl Acad. Sci. USA **115**, 1883-1888. (10.1073/pnas.1714478115) 29432166 PMC5828591

[5] Murray JM, Kaufmann GR, Hodgkin PD, Lewin SR, Kelleher AD, Davenport MP, Zaunders JJ. 2003 Naive T cells are maintained by thymic output in early ages but by proliferation without phenotypic change after age twenty. Immunol. Cell Biol. **81**, 487-495. (10.1046/j.1440-1711.2003.01191.x) 14636246

[6] Sottini A, Serana F, Bertoli D, Chiarini M, Valotti M, Vaglio Tessitore M, Imberti L. 2014 Simultaneous quantification of T-cell receptor excision circles (TRECs) and K-deleting recombination excision circles (KRECs) by real-time PCR. J. Vis. Exp. **94**, e52184. (10.3791/52184) PMC4396956 25549107

[7] Montecino-Rodriguez E, Berent-Maoz B, Dorshkind K. 2013 Causes, consequences, and reversal of immune system aging. J. Clin. Invest. **123**, 958-965. (10.1172/JCI64096) 23454758 PMC3582124

[8] Huang AT et al. 2020 A systematic review of antibody mediated immunity to coronaviruses: antibody kinetics, correlates of protection, and association of antibody responses with severity of disease. medRxiv 2020.04.14.20065771. (10.1101/2020.04.14.20065771) PMC7499300 32943637

[9] Wu C et al. 2020 Risk factors associated with acute respiratory distress syndrome and death in patients with coronavirus disease 2019 pneumonia in Wuhan, China. JAMA Intern. Med. **180**, 934-943. (10.1001/jamainternmed.2020.0994) 32167524 PMC7070509

[10] Sohrabi C, Alsafi Z, O'Neill N, Khan M, Kerwan A, Al-Jabir A, Iosifidis C, Agha R. 2020 World Health Organization declares global emergency: a review of the 2019 novel coronavirus (COVID-19). Int. J. Surg. **76**, 71-76. (10.1016/j.ijsu.2020.02.034) 32112977 PMC7105032

[11] Varatharaj A et al. 2020 Neurological and neuropsychiatric complications of COVID-19 in 153 patients: a UK-wide surveillance study. Lancet Psychiatry **7**, 875-882. (10.1016/S2215-0366(20)30287-X) 32593341 PMC7316461

[12] Chen Y et al. 2020 The novel severe acute respiratory syndrome Coronavirus 2 (SARS-CoV-2) directly decimates human spleens and lymph nodes. medRxiv 2020.03.27.20045427. (10.1101/2020.03.27.20045427)

[13] Tan L, Yang Y, Hong W, Huang M, Wu M, Zhao X. 2020 Lymphopenia predicts disease severity of COVID-19: a descriptive and predictive study. Signal Transduct. Target. Ther. **5**, 1-3. (10.1038/s41392-019-0089-y) 32296069 PMC7100419

[14] Mathew D et al. 2020 Deep immune profiling of COVID-19 patients reveals distinct immunotypes with therapeutic implications. Science **369**, eabc8511. (10.1126/science.abc8511) 32669297 PMC7402624

[15] Harrison C. 2020 Coronavirus puts drug repurposing on the fast track. Nat. Biotechnol. **38**, 379-381. (10.1038/d41587-020-00003-1) 32205870

[16] Rao VUS, Arakeri G, Subash A, Rao J, Jadhav S, Suhail Sayeed M, Rao G, Brennan PA. 2020 COVID-19: loss of bridging between innate and adaptive immunity? Med. Hypotheses **144**, 109861. (10.1016/j.mehy.2020.109861) 32505066 PMC7245229

[17] Folegatti PM et al. 2020 Safety and immunogenicity of the ChAdOx1 nCoV-19 vaccine against SARS-CoV-2: a preliminary report of a phase 1/2, single-blind, randomised controlled trial. The Lancet **396**, 467-478. (10.1016/S0140-6736(20)31604-4) PMC7445431 32702298

[18] Sekine T et al. 2020 Robust T cell immunity in convalescent individuals with asymptomatic or mild COVID-19. bioRxiv 2020.06.29.174888. (10.1101/2020.06.29.174888) PMC7427556 32979941

[19] Newman TJ. 2020 *Correlations in US COVID-19 mortality age profiles: epidemic start dates, geography and the PCF hypothesis*. Zenodo. (10.5281/zenodo.3976802)

[20] Salje H et al. 2020 Estimating the burden of SARS-CoV-2 in France. Science **369**, 208-211. (10.1126/science.abc3517) 32404476 PMC7223792

[21] Davies NG, Klepac P, Liu Y, Prem K, Jit M, Eggo RM. 2020 Age-dependent effects in the transmission and control of COVID-19 epidemics. Nat. Med. **26**, 1205-1211. (10.1038/s41591-020-0962-9) 32546824

[22] Rousseau MA, Chindelevitch L, An G, Hu L, Thareja R, Stephens D, Rish I. 2020 Understanding the thymus with applications to SARS-CoV-2 pathophysiology and susceptibility with potential therapeutics. Preprint.

[23] Zhang J et al. 2020 Changes in contact patterns shape the dynamics of the COVID-19 outbreak in China. Science **368**, 1481-1486. (10.1126/science.abb8001) 32350060 PMC7199529

[24] Bi Q et al. 2020 Epidemiology and transmission of COVID-19 in 391 cases and 1286 of their close contacts in Shenzhen, China: a retrospective cohort study. Lancet Infect. Dis. **20**, 911-919. (10.1016/S1473-3099(20)30287-5) 32353347 PMC7185944

[25] Metcalf CJE et al. 2021 Comparing the age and sex trajectories of SARS-CoV-2 morbidity with other respiratory pathogens points to potential immune mechanisms. medRxiv 2021.01.07.21249381. (10.1101/2021.01.07.21249381) PMC9198511 35719888

[26] Dorak MT, Karpuzoglu E. 2012 Gender differences in cancer susceptibility: an inadequately addressed issue. Front. Genet. **3**, 268. (10.3389/fgene.2012.00268) 23226157 PMC3508426

[27] Debacker M, Aguiar J, Steunou C, Zinsou C, Meyers WM, Scott JT, Dramaix M, Portaels F. 2004 Mycobacterium ulcerans disease: role of age and gender in incidence and morbidity. Trop. Med. Int. Health **9**, 1297-1304. (10.1111/j.1365-3156.2004.01339.x) 15598261

[28] Guerra-Silveira F, Abad-Franch F. 2013 Sex bias in infectious disease epidemiology: patterns and processes. PLoS ONE **8**, e62390. (10.1371/journal.pone.0062390) 23638062 PMC3634762

[29] Cook MB, McGlynn KA, Devesa SS, Freedman ND, Anderson WF. 2011 Sex disparities in cancer mortality and survival. Cancer Epidemiol. Biomark. Prev. **20**, 1629-1637. (10.1158/1055-9965.EPI-11-0246) PMC3153584 21750167

[30] Coggon D, Croft P, Cullinan P, Williams A. 2020 Assessment of workers personal vulnerability to covid-19 using covid-age. medRxiv 2020.05.21.20108969. (10.1101/2020.05.21.20108969) PMC7454792 32761080

[31] Waters A-M, Trinh L, Chau T, Bourchier M, Moon L. 2013 Latest statistics on cardiovascular disease in Australia. Clin. Exp. Pharmacol. Physiol. **40**, 347-356. (10.1111/1440-1681.12079) 23517328

[32] Nickbakhsh S, Ho A, Marques DFP, McMenamin J, Gunson RN, Murcia PR. 2020 Epidemiology of seasonal coronaviruses: establishing the context for the emergence of coronavirus disease 2019. J. Infect. Dis. **222**, 17-25. (10.1093/infdis/jiaa185) 32296837 PMC7184404

[33] Kissler SM, Tedijanto C, Goldstein E, Grad YH, Lipsitch M. 2020 Projecting the transmission dynamics of SARS-CoV-2 through the postpandemic period. Science **368**, 860-868. (10.1126/science.abb5793) 32291278 PMC7164482

[34] Cohen SA, Kellogg C, Equils O. 2020 Neutralizing and cross-reacting antibodies: implications for immunotherapy and SARS-CoV-2 vaccine development. Hum. Vaccines Immunother. **17**, 84-87. (10.1080/21645515.2020.1787074) PMC7872068 32678695

[35] Cowling BJ et al. 2012 Increased risk of noninfluenza respiratory virus infections associated with receipt of inactivated influenza vaccine. Clin. Infect. Dis. **54**, 1778-1783. (10.1093/cid/cis307) 22423139 PMC3404712

[36] Tsagarakis NJ, Sideri A, Makridis P, Triantafyllou A, Stamoulakatou A, Papadogeorgaki E. 2018 Age-related prevalence of common upper respiratory pathogens, based on the application of the FilmArray Respiratory panel in a tertiary hospital in Greece. Medicine **97**, e10903. (10.1097/MD.0000000000010903) 29851817 PMC6392546

[37] Ng KW et al. 2020 Pre-existing and de novo humoral immunity to SARS-CoV-2 in humans. bioRxiv 2020.05.14.095414. (10.1101/2020.05.14.095414) PMC7857411 33159009

[38] Grifoni A et al. 2020 Targets of T cell responses to SARS-CoV-2 coronavirus in humans with COVID-19 disease and unexposed individuals. Cell **181**, 1489-1501.e15. (10.1016/j.cell.2020.05.015) 32473127 PMC7237901

[39] Prabhakar M, Ershler EB, Longo DL. 2009 Bone marrow, thymus and blood: changes across the lifespan. Aging Health **5**, 385-393. (10.2217/ahe.09.31) 20072723 PMC2805199

[40] Kellogg C, Equils O. In press. The role of the thymus in COVID-19 disease severity: implications for antibody treatment and immunization. Hum. Vaccines Immunother. (10.1080/21645515.2020.1818519) PMC7993178 33064620

[41] Blank CU et al. 2019 Defining ‘T cell exhaustion’. Nat. Rev. Immunol. **19**, 665-674. (10.1038/s41577-019-0221-9) 31570879 PMC7286441

[42] Zheng M, Gao Y, Wang G, Song G, Liu S, Sun D, Xu Y, Tian Z. 2020 Functional exhaustion of antiviral lymphocytes in COVID-19 patients. Cell. Mol. Immunol. **17**, 533-535. (10.1038/s41423-020-0402-2) 32203188 PMC7091858

[43] De Biasi S et al. 2020 Marked T cell activation, senescence, exhaustion and skewing towards TH17 in patients with COVID-19 pneumonia. Nat. Commun. **11**, 3434. (10.1038/s41467-020-17292-4) 32632085 PMC7338513

[44] Diao B et al. 2020 Reduction and functional exhaustion of T cells in patients with coronavirus disease 2019 (COVID-19). medRxiv 2020.02.18.20024364. (10.1101/2020.02.18.20024364) PMC7205903 32425950

[45] Malandro N et al. 2016 Clonal abundance of tumor-specific CD4^+^ T cells potentiates efficacy and alters susceptibility to exhaustion. Immunity **44**, 179-193. (10.1016/j.immuni.2015.12.018) 26789923 PMC4996670

[46] Moore JB, June CH. 2020 Cytokine release syndrome in severe COVID-19. Science **368**, 473-474. (10.1126/science.abb8925) 32303591

[47] Desdín-Micó G et al. 2020 T cells with dysfunctional mitochondria induce multimorbidity and premature senescence. Science **368**, 1371-1376. (10.1126/science.aax0860) 32439659 PMC7616968

[48] Sudre CH et al. 2020 Attributes and predictors of long-COVID: analysis of COVID cases and their symptoms collected by the Covid Symptoms Study App. medRxiv 2020.10.19.20214494. (10.1101/2020.10.19.20214494)

